# Mapping periplasmic binding protein oligosaccharide recognition with neutron crystallography

**DOI:** 10.1038/s41598-022-20542-8

**Published:** 2022-10-21

**Authors:** Shantanu Shukla, Dean A. Myles, Matthew J. Cuneo

**Affiliations:** 1 Neutron Scattering Division, Oak Ridge National Laboratory, Oak Ridge, TN 37831 USA; 2 UT-ORNL Graduate School in Genome Science and Technology, The University of Tennessee at Knoxville, Knoxville, TN 37966 USA; 3 Department of Microbiology and Immunology, Northwestern University, Chicago, IL 60611 USA; 4 Department of Structural Biology, St. Jude Children’s Research Hospital, Memphis, TN 38105 USA

**Keywords:** X-ray crystallography, Carbohydrates, Molecular biophysics

## Abstract

Numerous studies have shown how periplasmic binding proteins (PBPs) bind substrates with exquisite specificity, even distinguishing between sugar epimers and anomers, or structurally similar ions. Yet, marked substrate promiscuity is also a feature encoded in some PBPs. Except for three sub-Ångström crystal structures, there are no reports of hydrogen atom positions in the remaining (> 1000) PBP structures. The previous X-ray crystal structure of the maltodextrin periplasmic-binding protein from *Thermotoga maritima* (tmMBP) complexed with oligosaccharide showed a large network of interconnected water molecules stretching from one end of the substrate binding pocket to the other. These water molecules are positioned to form multiple hydrogen bonds, as well as forming interactions between the protein and substrate. Here we present the neutron crystal structure of tmMBP to a resolution of 2.1 Å. This is the first neutron crystal structure from the PBP superfamily and here we unambiguously identify the nature and orientation of the hydrogen bonding and water-mediated interactions involved in stabilizing a tetrasaccharide in the binding site. More broadly, these results demonstrate the conserved intricate mechanisms that underlie substrate-specificity and affinity in PBPs.

## Introduction

Periplasmic binding proteins (PBPs) are the ligand-binding components of ATP-binding cassette (ABC) transporters and are sometimes associated with chemotaxis systems. Members of the PBP superfamily mediate the uptake and transmembrane transport of a diversity of metabolically important solutes in bacteria, such as carbohydrates, ions, amino acids, and polyamines, to name a few.

Despite the wide variation in PBP ligand size and chemical functionality the two domain PBPs have a conserved αβ-fold, with each domain encompassing a central β-stranded core that is sandwiched between surrounding α-helices^1^. Ligand binding at the interdomain interface induces a hinge-bending motion in which the two solvent exposed polar interfaces of each domain close down on and envelop ligands in a largely desolvated binding site reminiscent of the protein interior^2–4^. However, in many instances water molecules remain within the binding site, and these solvent interactions with ligands have been shown to be important in broadening PBP substrate specificity or fine tuning highly specific binding^5,6^. For example, in PBPs that share the oligopeptide-binding protein fold, water molecules have been shown to act as adapters, matching the ligand hydrogen-bonding requirements and permitting a diverse array of substrates to be accommodated within a single binding site^7,8^. In some cases, rather than serving to increase promiscuity, water molecules are also used in PBPs to impart specificity beyond what can be encoded within the repertoire of protein backbone and side-chains^6,9^. In yet others, PBPs have been shown to have bipartite binding sites, where each sub-site differentially uses water to impart either specificity or promiscuity^2,10^. Taken together, the diversity of PBP binding site adaptation mechanisms suggests that determining water molecule orientation and hydrogen bonding patterns within the PBP substrate binding site is indispensable for understanding the fine tuning of ligand recognition within this class of proteins.

More than 1000 crystal structures of PBPs have been determined thus far. Of these, only three were determined at sub-Ångstrom resolution; glucose-binding protein (PDB ID:2FVY)^11^, glutamine-binding protein (PDB ID: 4KQP)^12^, and a phosphate-binding protein (PDB ID: 4F1V)^13^. In these three crystal structures, the resolution of the diffraction data permitted the explicit positioning of many protein hydrogen atoms, yet no hydrogen atoms could be modeled on solvent molecules. In all others, the position of key hydrogen atoms and patterns of hydrogen bonding interactions within the PBP binding site are inferred, rather than experimentally determined. Here we report the first neutron crystal structure of a PBP, namely a maltodextrin-binding protein (MBP) from a cluster of three related proteins found in *Thermotoga maritima* (tmMBP)^14^. This study reveals the explicit hydrogen bonding interactions and water network within the substrate binding site at an unprecedented level of detail, lending valuable insights into the intricate mechanisms that underpin the derivation of substrate-specificity and affinity in PBPs.

## Results

### Neutron structure of maltotetraose bound tmMBP

The genome of *T. maritma* encodes for three isoforms of maltose binding proteins; the tmMBP2 isoform was used in the present study and is called tmMBP going forwards. Large crystals of tmMBP that were suitable for neutron diffraction experiments were grown in sitting drops using nine-well siliconized glass plates. Each sitting drop was made by mixing 400 μl of protein with an equal volume of the mother liquor supplemented with 10% (vol./vol.) deuterated glycerol to slow the rate of crystallization and growth, and incubated at 20 °C. Large crystals (3–10 mm^3^) appeared within 30 days and were transferred weekly into sandwich boxes with fresh deuterated mother liquor to promote replacement of exchangeable hydrogen atoms for deuterium. A total of four mother liquor exchanges were performed. Crystals were transferred to quartz capillaries with a D_2_O mother liquor plug and sealed with wax for room temperature neutron and X-ray data collection.

The room temperature X-ray crystal structure of the tmMBP complexed with maltotetraose was determined at 1.70 Å with an R_work_/R_free_ of 14.1/17.5%. The X-ray model contained a total of 3099 non-hydrogen atoms (C, N, O, S), of which 2893 were protein atoms, 45 ligand atoms, and 161 solvent atoms. Prior to joint refinement, hydrogen atoms were added to the structure, where exchangeable positions had mixed hydrogen/deuterium occupancy. This starting model was used for joint X-ray/neutron refinement in Phenix^15,16^ where H/D occupancy and position was experimentally modelled based on the neutron diffraction data. The final room temperature neutron structure was determined to a resolution of 2.10 Å and refined to an R_work_/R_free_ of 24.3/27.3%. Data collection statistics and details on the final refined model are given in Table 1.

**Table 1:** Data collection and refinement statistics.

	Neutron	X-ray
Wavelength	2.78–4.50 Å	1.54 Å
Resolution range^a^	35.71–2.10 (2.21–2.10)	89.75–1.70 (1.76–1.70)
Space group	P2_1_	
Unit cell	a = 35.91 b = 56.33 c = 90.00 β = 94.3	
Total reflections	52,954 (4675)	786,386 (72,561)
Unique reflections	15,172 (1628)	39,602 (3961)
Multiplicity	3.5 (2.9)	19.9 (18.3)
Completeness (%)	73.1 (54.2)	100.00 (100.00)
Mean I/sigma(I)	7.40 (1.70)	51.66 (9.85)
Wilson B-factor	7.73	18.65
R_merge_	0.144 (0.300)	0.124 (0.378)
R_meas_	0.165 (0.347)	0.12 (0.40)
R_pim_	0.078 (0.168)	0.02 (0.09)
CC_1/2_	NA	0.99 (0.96)

Joint neutron/X-ray refinement		
Reflections used in refinement	15,145 (1055)	39,571 (3957)
Reflections used for R_free_	730 (96)	1932 (128)
R_work_ (%)	24.5 (32.7)	14.1 (17.7)
R_free_ (%)	27.2 (37.1)	17.5 (21.9)
Number of hydrogen atoms	3829	
Number of non-hydrogen atoms	3081	
Macromolecules	2875	
Ligands	45	
Solvent	161	
R.M.S. bonds (Å)	0.014	
R.M.S. angles (°)	1.2	
Ramachandran favored (%)	99.2	
Ramachandran allowed (%)	0.8	
Ramachandran outliers (%)	0	
Rotamer outliers (%)	3.0	
Clashscore	3.3	
Average B-factor	25.0	
Macromolecules	24.3	
Ligands	17.3	
Solvent	39.8	

^a^Values in parentheses represent the highest resolution shell.

**Figure 1:**
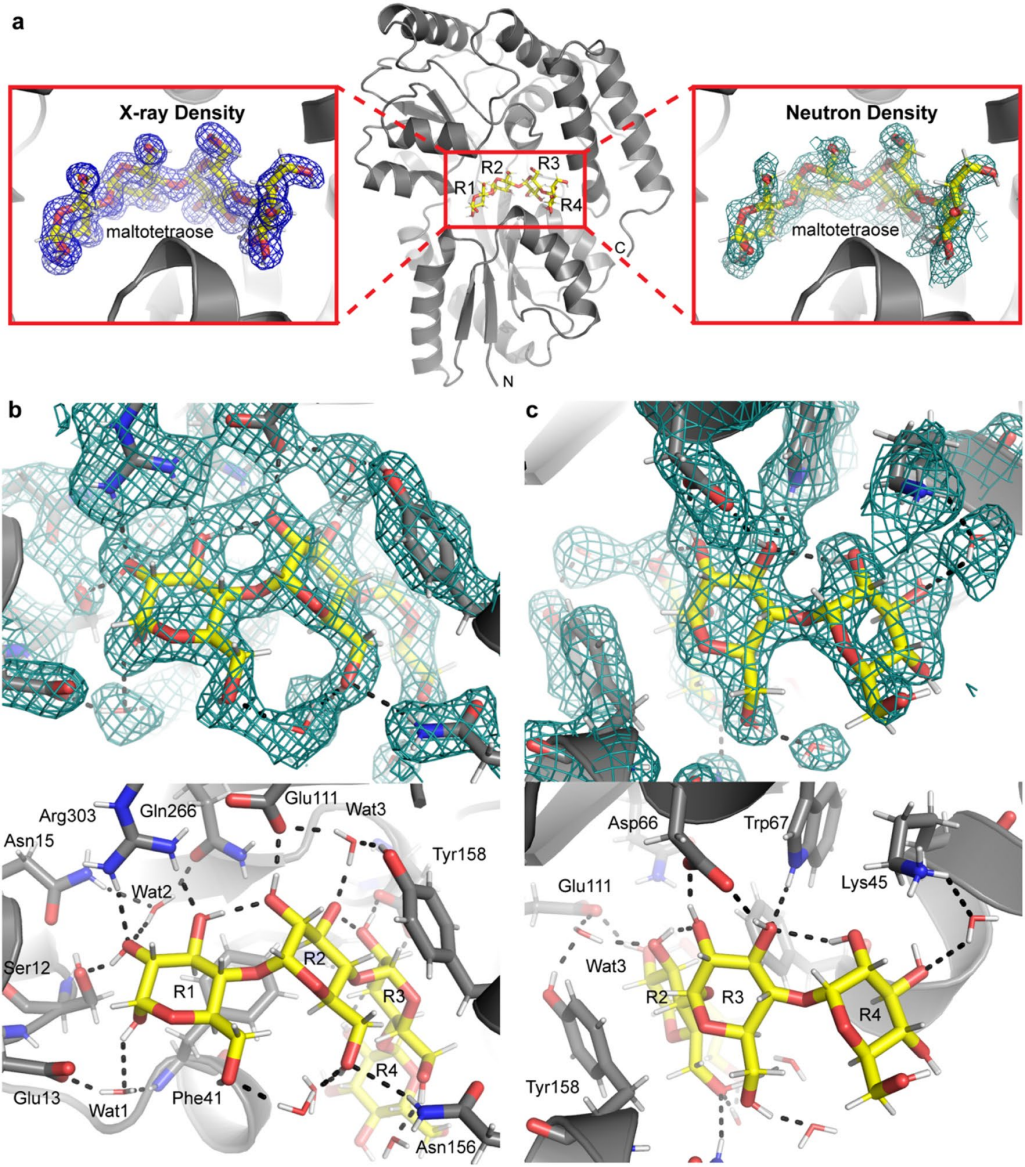
The neutron crystal structure of tmMBP. (**a**) Center, overall structure of tmMBP bound to maltotetraose (stick representation with yellow carbon atoms). The N- and C-termini are indicated as well as the nomenclature for the maltotetraose rings (R1–R4). Left, 2Fo–Fc electron density for maltotetraose contoured at 1.4 sigma. Right, 2Fo–Fc nuclear density for maltotetraose contoured at 1.1 sigma. (**b**) Top, close-up view of the 2Fo–Fc nuclear density for the R1/R2 subsite contoured at 1.0 sigma. Bottom, close-up view of the R1/R2 hydrogen bonding network identified from the nuclear density maps. Hydrogen bonds are represented as black dashed lines. (**c**) Top, close-up view of the 2Fo–Fc nuclear density for the R3/R4 subsite 1.0 sigma. Bottom, close-up view of the R3/R4 hydrogen bonding network identified from the nuclear density maps. Hydrogen bonds are represented as black dashed lines.

Overall, the room temperature 2.1 Å neutron structure of tmMBP (Fig. 1) is very similar (r.m.s.d. = 0.36 on backbone atoms) to the published X-ray structure determined at cryogenic temperatures (PDB ID: 6DTS)^14^. The neutron structure enabled the modelling of deuterium atoms at exchangeable sites on the protein and the bound ligand, as well as deuterium atoms on solvent water molecules. The nuclear density maps showed clearly resolved density for the maltotetraose substrate in the binding site (Fig. 1). Importantly, the neutron structure also resolves the position and orientation of D_2_O solvent molecules within the substrate binding cavity, which were previously inferred to provide hydrogen bonding interactions in the earlier cryogenic X-ray model (Fig. 1). Thus, the neutron structure shows directly how the network of water molecules that traverse the entire length of the binding cavity contribute to and mediate hydrogen bonding interactions between the protein and substrate.

### Hydrogen bonding interactions in the tmMBP maltotetraose binding site

The tmMBP binding site uses a network of polar amino acids to satisfy the hydrogen bonding potential of many of the maltotetraose hydroxyl groups (Fig. 1b,c). Yet, a distinct pattern of hydroxyl group recognition is observed depending on where the carbohydrate ring is situated within the tmMBP binding site. A total of 8 specific hydrogen bonds, defined here as mediated either through direct protein/carbohydrate hydrogen bonds or through directly-bound water molecules, are observed for the first two sugar rings (ring 1/R1 and ring 2/R2) (Fig. 1b). The hydroxyl moieties of the first two sugar rings are all coordinated by specific hydrogen bonds, apart from the R1 O6 hydroxyl. The hydrogen bond formed between Glu111 and the R2 O2 hydroxyl is a short hydrogen bond^17^, with 2.7 Å between heavy atoms, yet the hydrogen atom is not found to be delocalized from a canonical position. A total of 4 specific hydrogen bonds are formed with the remaining two rings (ring 3/R3 and ring 4/R4) (Fig. 1c). The hydrogen bond formed between Asp66 and the ring 3 O2 hydroxyl is also a short hydrogen bond, with 2.7 Å between heavy atoms, yet it is also a canonical hydrogen bond.

**Figure 2:**
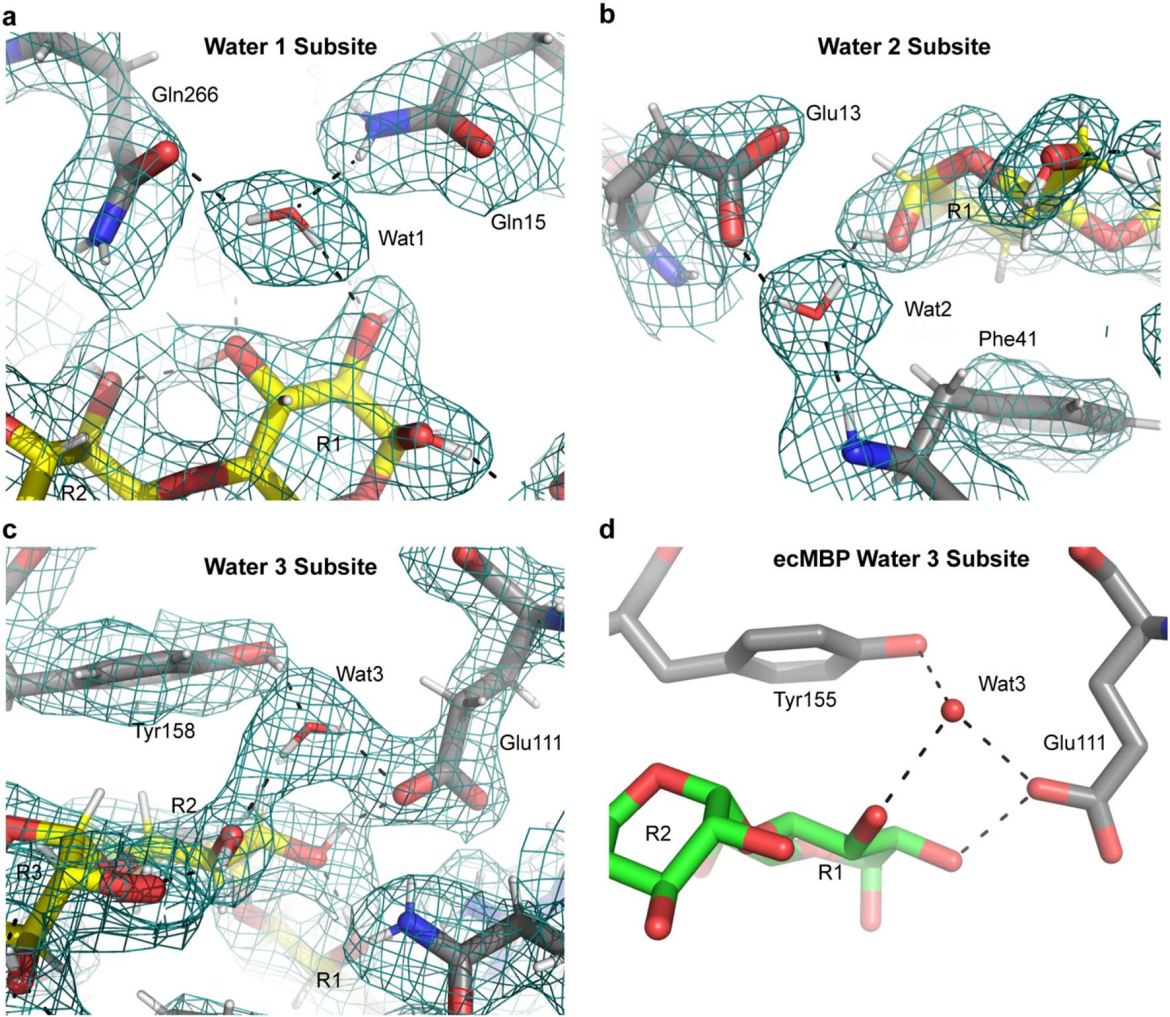
Water structure in the tmMBP binding site. (**a**) tmMBP water subsite 1. (**b**) tmMBP water subsite 2. (**c**) tmMBP water subsite 3. (**d**) ecMBP water subsite 3. Protein sidechains are represented as sticks with grey carbon atoms, maltotetraose is represented as sticks with yellow carbon atoms, and maltose is represented as sticks with green carbon atoms. Hydrogen bonds are indicated as black dashed lines. Nuclear density is show for the tmMBP water subsites and is contoured at 1.0 sigma.

The differential hydrogen bonding pattern of the R1/R2 and R3/R4 subsites suggests the tmMBP has a bipartite binding site. The bulk (8/12 hydrogen bonds) of the specific interactions occur in the “specificity subsite” surrounding the R1/R2 carbohydrate rings, whereas the remaining subsite leaves 4 of the hydroxyl groups with no specific hydrogen bonds (Fig. 1c). The water molecules bound in the specificity subsite are responsible for forming 3 of 8 hydrogen bonds and are thus important in binding of saccharides in tmMBP. The neutron diffraction data allows for directly orienting the water molecules rather than inferring their positions and hydrogen bonding pattern (Fig. 2). Each water molecule forms three hydrogen bonds, two with the binding pocket side chains and one with maltotetraose (Fig. 2). These water molecules all have lower than average b-factors (average of 20.7 Å^2^) from the remainder of the other solvent molecules (average of 40.5 Å^2^). Indeed, water three has the lowest b-factor of all solvent molecules. Interestingly, although the canonical MBP, *Escherichia coli* maltose-binding protein (ecMBP), binds maltodextrins in a shifted register (where tmMBP ring 2 corresponds to the ecMBP ring 1), the amino acids coordinating water 3, and the hydrogen bonds formed, are conserved as in tmMBP (Fig. 2d)^18^. Moreover, water 3 is also the solvent molecule with the lowest b-factor in ecMBP^18^. Taken together, these observations highlight the importance of the water network in the tuning the specificity of MBPs in general.

## Discussion

Neutron crystallography is a powerful tool for determining hydrogen and water orientations in a protein structure^19^. Despite the wealth of knowledge underlying the fine-tuning of molecular recognition in the PBP superfamily, the 2.1 Å neutron structure of tmMBP is the first neutron crystal structure of any PBP. The bipartite tmMBP binding sight holds a large number of water molecules. Some of these have a direct role in mediating substrate specificity and are thus important for tight binding and specific recognition of saccharides. This contrasts with the paradigmatic role of the entropic contribution of water molecules to ligand binding affinity. It was recently shown that perturbing the PBP water network can significantly alter substrate-binding affinity, suggesting that binding pocket solvent molecules serve as an evolutionary constraint to maintain and modulate the affinity of substrate interactions^6^. Indeed the present work demonstrates the conservation of this concept in MBPs and thus reinforces the fundamental importance of water molecules in tuning PBP specificity^6,20^.

## Methods

### Expression, purification, and crystallization of tmMBP

For the neutron structure of tmMBP, unlabeled tmMBP was expressed and subjected to H/D exchange as described in the Results section. Unlabeled tmMBP was recombinantly expressed in *E. coli* and purified as previously described^14^. Briefly, Enfors minimal media expressed tmMBP was purified in a two-step chromatographic process, consisting of an initial nickel affinity chromatography column followed by size exclusion chromatography. The purified protein was extensively dialyzed in 20 mM Tris pH 7.5 and 40 mM NaCl, concentrated to 25 mg/ml, and crystallized using previously optimized conditions of 24–25% (wt./vol.) PEG 3350, 0.2 M sodium acetate, 0.1 M BisTris pH 5.5 in H_2_O solution^14^.

### X-ray and neutron diffraction data collection and joint refinement

Neutron diffraction data were collected on the IMAGINE neutron diffractometer at the High Flux Isotope Reactor, Oak Ridge National Laboratory^21–23^. A total of 35 quasi-Laue diffraction images were collected (λ = 2.78–4.50 Å) at room temperature, with exposure time of 15.5 h per frame. Data were collected at two crystals settings, with the crystal rotated 10° on φ between successive images. Diffraction images were processed and integrated using the LAUEGEN package^24,25^. Wavelength normalization and scaling between Laue diffraction images was performed using LSCALE. The combination of low crystal symmetry and the cylindrical detector array limited overall neutron data completeness to ~ 73%, and to 51% in the highest 2.1 Å resolution shell (Table 1). The same crystal used for neutron diffraction was then used to collect room temperature X-ray diffraction dataset on a Rigaku 007HF diffractometer with an a EIGER R 4 M hybrid photon counting detector. Data were collected using the strategy mode in *CryAlis*^*Pro*^ (Rigaku. The Woodlands, Texas), with exposure times of 1 s per 0.25° phi-rotation and omega scans of 54–78° at 20 crystal settings. The data were integrated using *CryAlis*^*Pro*^ and reduced and scaled in *AIMLESS*^26^.The X-ray data extended to 1.70 Å and was 100% complete (Table 1). The previously determined structure of tmMBP (PDB code 6DTS) was used for phasing in Phaser^15^. Joint X-ray and neutron refinement was performed using PHENIX Refine^16,27^ and manual model building of the single chain found in the asymmetric unit of tmMBP was carried out in COOT^28^.

## Data Availability

The X-ray and neutron structure factors and the final refined atomic model for maltotetraose bound tmMBP have been deposited to the Protein Data Bank under accession code 8DHD.
